# Appendicular Skeletal Muscle Mass (ASMM) and Fat-Free Mass (FFM) DXA–BIA Estimations for the Early Identification of Sarcopenia/Low Muscle Mass in Middle-Aged Women

**DOI:** 10.3390/nu16223897

**Published:** 2024-11-15

**Authors:** Alessia Moroni, Clara Gasparri, Simone Perna, Mariangela Rondanelli, Margherita Micheletti Cremasco

**Affiliations:** 1 Department of Life Science and Systems Biology, University of Torino, Via Accademia Albertina, 13, 10123 Torino, Italy; alessia.moroni@unito.it; 2 Endocrinology and Nutrition Unit, Azienda di Servizi alla Persona “Istituto Santa Margherita”, University of Pavia, 27100 Pavia, Italy; clara.gasparri01@universitadipavia.it; 3 Department of Food, Environmental and Nutritional Sciences, Division of Human Nutrition, Università Degli Studi di Milano, 20133 Milan, Italy; simoneperna@hotmail.it; 4 Department of Public Health, Experimental and Forensic Medicine, University of Pavia, 27100 Pavia, Italy; mariangela.rondanelli@unipv.it

**Keywords:** DXA, BIA, appendicular skeletal muscle mass, fat-free mass, sarcopenia

## Abstract

Background/Objectives: Sarcopenia involves the loss of muscle mass along with a decrease in muscle strength and physical performance. The aim of this paper was to compare the already published BIA equations for the estimation of Appendicular Skeletal Muscle Mass (ASMM) and Fat-Free Mass (FFM) with dual X-ray densitometer DXA estimations in order to determine whether Bioelectrical Impedance Analysis (BIA) could be a feasible application on a general population for the detection of low muscle mass and sarcopenia. Methods: Seventy-nine healthy women aged 40–70 years were included. Assessments involved BIA and DXA evaluations and anthropometric measurements. Results: DXA and BIA estimations showed great agreement, particularly the ones introduced by Scafoglieri et al. (2017) for ASMM (mean difference 1.81 kg) and Kanellakis et al. (2020) equation for FFM (mean difference 0.52 kg) resulted in the best fit for the cohort in analysis. BIA could intercept a low muscle mass condition which can be linked to sarcopenia. Conclusions: This study showed how the use of BIA represents an effective and reliable method in the evaluation of sarcopenia.

## 1. Introduction

Sarcopenia was defined in 1997 by Rosenberg et al. (1997) [1] as the age-related loss of skeletal muscle mass that decreases muscle strength, function, and quality of life. The ultimate definition of such disease states that sarcopenia “comprises the concurrent combination of reduced muscle mass and muscle strength”. Moreover, authors of the Delphi Consensus from the Global Leadership Initiative in Sarcopenia (GLIS) found that sarcopenia is a generalised disease of skeletal muscle, and prevalence increases with age while the condition is a potentially reversible disease [2]. The overall estimates of prevalence in the world in 2017 was 10% (95% CI: 8–12%) in men and 10% (95% CI: 8–13%) in women, respectively [3], and a further increase is expected (i.e., prevalence could increase by 72.4% in 2045 in subjects aged 65 years and older) [4]. Sarcopenia, according to a recent review, is believed to affect 13% of individuals around the world [5]. In all cases, using different classifications and cut-off points, it appears from the literature that the prevalence of sarcopenia varies between 10% and 27% in the world [6]. Compared to peak values in their early and late 30s, people in their 70s lose 30% of their muscle strength, although the process is already noticeable by the age of 40–50 [7,8,9]. Therefore, it should be noted that loss of muscle mass and strength is not limited to late age [7]. In fact, skeletal muscle loss begins at 35 years of age, but after the age of 65, the decrease becomes significant (muscle loss increases to 3% per year), leading to debilitation in senescence [10]. If, however, this decrease exceeds the physiological trend, screening and preventive interventions should be considered, as the guidelines provided by the European Working Group on Sarcopenia in Older People [9] affirmed how it is imperative to tackle this issue starting at the age of 50. This involves early identification of signs of excessive loss of muscle mass in relation to age. To date, the diagnostic process is mostly performed in the clinical setting and geared toward the over-60 age group. Specifically, women in menopause are very sensitive to muscle mass decrease, mainly due to the reduction in oestrogens, which is linked to a decline in protein synthesis. Moreover, during menopause, chronic inflammation and oxidative stress increase, also impacting muscle mass and contributing to its loss [11,12]. Concerning lifestyle, women in menopause tend to exercise less, and the lack of movement, along with the abovementioned factors, can lead to great muscle reduction [11].

In order to identify and confirm sarcopenia, the most recent revision of the recommendations [9] suggested an algorithm (called Find-Assess-Confirm-Severity, or F-A-C-S) that includes particular steps, but the diagnosis can only be confirmed by the discovery of decreased muscle mass. A number of methods can be employed to perform this assessment: the most popular ones are X-ray densitometry (DXA) and bioelectrical impedance analysis (BIA) using population specific pre-validated equations, in addition to the gold standards that are not universally applicable (i.e., Magnetic Resonance Imaging (MRI), Computer Tomography (CT)). In particular, appendicular skeletal muscle mass (ASMM) is the main focus in terms of muscle amount. In addition, regarding muscle quality, there is still no consensus in the literature as to which technique/methodology is most appropriate for such assessment. In this regard, one of the variables derived from bioelectrical impedance analysis is the phase angle, which is mentioned in the European Working Group on Sarcopenia in Older People 2 (EGSWOP2) [9] as a factor worthy of further study and research, given its speed, low cost, and easy applicability.

In the primary prevention perspective, the ultimate aim is to detect the risk of sarcopenia early through population screenings that can be easily applied and managed in contexts like Workplace Health Promotion Programmes (WHPPs), involving activities such as health education and education on correct nutrition as well as healthy lifestyle habits, but also in public domains such as university Third Mission initiatives or screenings held at social events or centres. The intent is to generate knowledge outside academic environments and to realise projects based on interaction among universities, industries, and society for the benefit of the community, as in the case of population screening for health and primary prevention. In such environments and settings, complex methods of screening like DXA are very difficult to apply due to issues with portability, costs, and invasiveness, while measurements and performance evaluation methods like the BIA can be used to screen a huge number of individuals. Indeed, because it is non-invasive and portable, the BIA approach proves to be quick, affordable, and broadly useful in several clinical and non-clinical settings [13,14].

For the purpose of providing a methodological contribution for the identification of sarcopenia indicators with effective, inexpensive, and reliable methods and to understand the effectiveness of BIA in the first phase of sarcopenia evaluation, this study reports data collected at Santa Margherita Hospital of Pavia (Pavia, Italy), which usually deals with sarcopenia assessment and treatment. Health professionals actively helped in conducting the project, fully collaborating with the University of Torino group. Concurrently, researchers introduced muscle evaluations and estimations through BIA using already published equations in order to compare them with estimations through DXA and thus determine whether BIA could be feasibly applied in a general population for the assessment of ASMM and FFM.

## 2. Materials and Methods

The study was conceived and conducted in collaboration between the universities of Torino and Pavia, and it was approved by the Bioethics Committee of the University of Torino in March 2023 (Protocol n.0214399; 6 April 2023). The research was conducted in accordance with the Declaration of Helsinki for human studies of the World Medical Association (World Medical Association, 2013). After receiving a thorough verbal explanation, participants had to sign an informed consent form. Considering the above-mentioned data regarding the onset age of muscle loss and in a prevention perspective, inclusion criteria involved healthy females aged between 40 and 70 years old, attending the Endocrinology and Nutrition Unit, Azienda di Servizi alla Persona “Istituto Santa Margherita”, for routine visits or screenings. Exclusion criteria were severe pathologies such as cancer, neurodegenerative diseases, neuromotor diseases, and mobility impairments as well as chronic kidney disease (CKD), cirrhosis, or participants with impactful ongoing therapies. Other conditions which did not involve muscle deterioration were considered acceptable. Participation was exclusively on a voluntary basis. Data collection took place at Istituto Santa Margherita (Pavia) from February 2023 to April 2024.

### 2.1. Assessments

With regard to the muscle mass evaluation, following the latest European EWGSOP2 guidelines [9], researchers assessed body composition in the following ways:

- Through DXA, by dual X-ray densitometer with the use of a Lunar Prodigy DXA (GE Medical Systems, Milwaukee, WI, USA). Such a method yields bone mineral content (BMC), lean mass, and appendicular lean soft tissue (ALST);
- Through BIA, using BIA 101 BIVA PRO bioelectrical impedance analyser (Akern Srl, Pontassieve, Italy) and Bodygram Dashboard^®^ Software, that besides other body compartments, yields the targeted body composition estimates such as fat- free mass (FFM) and appendicular skeletal muscle mass (ASMM). The technique involves the volunteer lying down for a few minutes, with their limbs slightly apart. The instrument, through electrodes placed on different areas of the body, injects a mild current of low intensity and constant frequency (250 µA and 50 kHz) that cannot be felt by the subject and is completely non-harmful (BIA 101 BIVA PRO Model Manual reference). Following standard methods established by the National Institutes of Health [15], each participant was requested to remove any metal objects and was then measured while wearing only pants, being careful to ensure participants had an empty bladder and skin free of oils or body lotions. Bioelectrical tissue values were measured on the right hemisoma between the ipsilateral wrist and anklebone prominences (metatarsus–metacarpus area) while the participants were lying on their backs on a medical non-conductive surface (bed). There was 5 cm separating each pair of electrodes.

Moreover, anthropometric measurements such as stature and weight were assessed following the ISO-7250-1 (2017) [16] international standards.

### 2.2. Comparison of DXA and BIA

Concerning the comparison between the two methods, researchers reviewed the literature in order to target the most used one, and thus validated BIA equations for the estimation of ASMM. Three equations were selected for comparison of the appendicular lean soft tissue mass (ALST) estimated with DXA:

- Kyle et al. (2003) [17] (246 men and 198 women aged 22–94 years) = −4.211 + (0.267 height^2^/resistance) + (0.095 × weight) +(1.909 × sex) + (−0.012 × age) + (0.058 × reactance) (men = 1, women = 0);
- Sergi et al. (2015) [18] (296 subjects over 60, mean age 71.4 ± 5.4) = −3.964 + (0.227 × RI) + (0.095 × weight) + (1.384 × sex) + (0.064 × Xc) (women = 0; men = 1);
- Scafoglieri et al. (2017) [19] (291 patients older than 65 years of age and if they had body mass index (BMI) between 20 and 30) = 1.821 + (0.168 × height^2^/resistance) + (0.132 × weight) + (0.017 × reactance) − (1.931 × sex) (women = 1; men = 0).
  In relation to the whole body, researchers compared the fat-free mass (FFM) obtained by DXA (Bone Mineral Content (BMC) + Lean Mass (LM)) with the estimation of fat-free mass (FFM) obtained by BIA through the following equations:
- Kanellakis et al. (2020) [20] (694 Greek adults, 429 women and 265 men aged 40.36 ± 15.221 years) FFM (kg) = 12.299 + (0.164 × Weight (kg)) + (7.287 × Gender) − (0.116 × Resistance (ohm)/Height (m)^2^) + (0.365 × Reactance (ohm)/Height (m)^2^) + (21.570 × Height (m)) (female = 0, male = 1);
- Bodygram Dashboard^®^ FFM equation (Akern Srl).

### 2.3. Statistical Analyses

Normality of data was assessed through Kolmogorov–Smirnov test and Shapiro–Wilk test. Data analyses were performed using SPSS version 29 and Microsoft Excel. Correlations were made through Pearson’s R test, while agreement between DXA and BIA was made through Bland–Altman plot and consequence regression analysis. Comparisons between groups were performed by means of Independent Student’s *t*-test. Statistical significance was accepted as *p* < 0.05.

## 3. Results

A total of 108 women participated in the study, but only 79 were eligible for the analyses. As muscle mass decreases during ageing, and mostly after the menopause period, researchers decided to firstly divide the sample by means of age (40–55 years old (n = 38) and 56–70 years old (n = 41)) and by means of presence of menopause status (Menopause (N = 56) and No menopause (N = 23)). Age of menopause ranged from 41 to 58, with a mean of 49.38 (±3.83).

### 3.1. Descriptive Analysis

Descriptive statistics are illustrated in Table 1 and Table 2. Despite the oriented decision to divide the sample, in the comparison of age, there are no significant differences between the groups for all the variables except for phase angle (PA) and obviously for age. In the comparison by means of menopause presence, researchers observed, again, a statistical difference for the phase angle but also for ALST, ASMM (Kyle et al., 2003) [17], and FFM [20]. This result might indicate better muscle quality in younger individuals and women who still have menstrual cycles, along with a higher quantity of muscle in the latter group. However, this last observation should be interpreted with caution, as other estimations did not reveal significant differences, despite values close to <0.05.

Although researchers divided the sample twice (by means of age and of menopause presence), the following analyses concerned the sample as a whole (N = 79) as there were not enough significant differences between the groups. Beyond the fact that the division on the basis of menopause yielded more differences, indicating a probable difference also in muscle quantity, we judged them not sufficient for separate analyses due to their numerical heterogeneity (N = 23 and N = 56).

### 3.2. Correlation Between DXA and BIA

As shown in Table 3, correlations between DXA and BIA estimations were performed with Pearson’s R correlation. With regard to the limbs, all of the correlations yielded very high R coefficients (close to 1, values higher than 0.80) and a statistical significance of *p* < 0.001 in all cases. In relation to the whole body, all the correlations yielded very high R (in this case, higher than 0.90). Such results indicated how the estimations from the already published BIA equations highly correlate to the DXA estimations.

### 3.3. DXA–BIA Agreement: Bland–Altman Plot

In order to better understand the agreement between DXA and the estimations obtained through BIA, researchers used a Bland–Altman plot and consequence regression analysis for all the already existing equations versus the estimations made through DXA.

#### 3.3.1. Kyle et al. (2003) and ALST (DXA)

**Figure 1:**
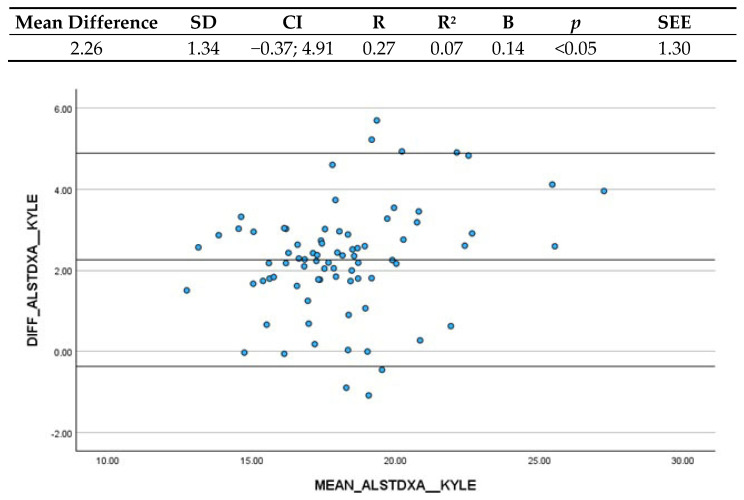
Bland–Altman plot and regression analysis between Kyle et al.’s (2003) [17] ASMM estimation and ALST estimated by DXA. SD = standard deviation; CI = Confidence Interval; R = correlation coefficient; R^2^ = correlation coefficient squared; B = regression coefficient; *p* = significance; SEE = standard estimation error.

Concerning the comparison between Kyle et al.’s (2003) [17] ASMM estimation and ALST estimated through DXA, the mean difference is 2.26 kg (CI: −0.37; 4.91), indicating that BIA underestimated DXA estimations of more than 2 kg (Figure 1). The Confidence Interval highlighted that 95% of the differences between the two methods fall between such ranges and was determined clinically acceptable. Moreover, researchers performed a linear regression analysis between the differences and the mean of the measurements. The coefficient B resulted in 0.14, and the significant *p*-value indicated that there is actually a systematic variation depending on the level of the measurement. Such variation is, however, very little and thus clinically acceptable.

#### 3.3.2. Sergi et al. (2015) and ALST (DXA)

**Figure 2:**
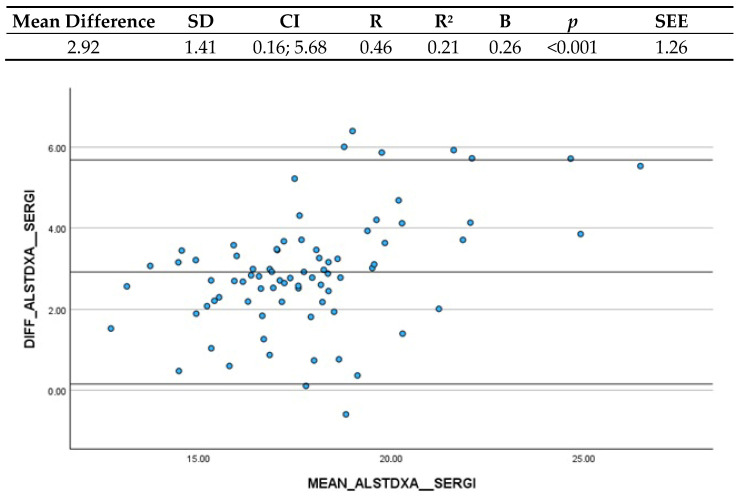
Bland–Altman plot and regression analysis between Sergi et al.’s (2015) [18] ASMM estimation and ALST estimated by DXA. SD = standard deviation; CI = Confidence Interval; R = correlation coefficient; R^2^ = correlation coefficient squared; B = regression coefficient; *p* = significance; SEE = standard estimation error.

The mean difference between Sergi et al.’s (2015) [18] ASMM estimation and ALST estimated though DXA is 2.92 kg (CI: 0.16; 5.68), indicating an underestimation of almost 3 kg through BIA measurements (Figure 2). The Confidence Interval was determined to be clinically acceptable. The linear regression analysis between the differences and the mean of the measurements yielded a B coefficient of 0.26, and the significant *p*-value indicated that there is a systematic variation depending on the level of the measurement. This variation is considered quite low and thus clinically acceptable.

#### 3.3.3. Scafoglieri et al. (2017) and ALST (DXA)

**Figure 3:**
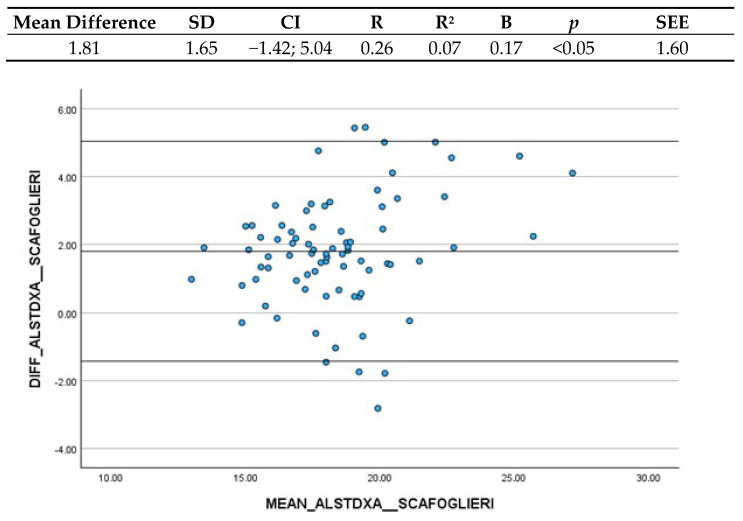
Bland–Altman plot and regression analysis between Scafoglieri et al.’s (2017) [19] ASMM estimation and ALST estimated by DXA. SD = standard deviation; CI = Confidence Interval; R = correlation coefficient; R^2^ = correlation coefficient squared; B = regression coefficient; *p* = significance; SEE = standard estimation error.

As shown in Figure 3, the mean difference between Scafoglieri et al.’s (2017) [19] ASMM estimation and ALST estimated through DXA is 1.81 kg (CI: −1.42; 5.04), indicating that BIA slightly underestimated DXA measurements. The Confidence Interval was determined to be clinically acceptable. The linear regression analysis between the differences and the mean of the measurements yielded a B coefficient of 0.17 and the significant *p*-value indicated that there is actually a systematic variation depending on the level of the measurement; however, it is very low and thus clinically acceptable.

#### 3.3.4. Kanellakis et al. (2020) and FFM (DXA)

**Figure 4:**
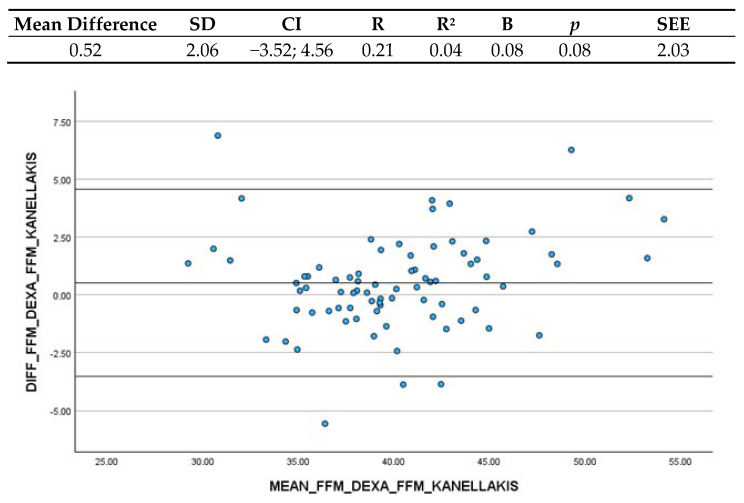
Bland–Altman plot and regression analysis between Kanellakis et al.’s (2020) [20] ASMM estimation and FFM estimated by DXA. SD = standard deviation; CI = Confidence Interval; R = correlation coefficient; R^2^ = correlation coefficient squared; B = regression coefficient; *p* = significance; SEE = standard estimation error.

With regard to the total body, and thus FFM, the comparison between Kanellakis et al.’s (2020) [20] FFM estimation and FFM obtained through DXA shows that the mean difference is very little: 0.52 kg (CI: −3.52; 4.56) (Figure 4). This indicates that BIA overestimated DXA measurements by just 0.5 kg. The Confidence Interval highlights that 95% of the differences between the two methods fall between such range and, despite being quite high, was determined clinically acceptable. Then, researchers performed a linear regression analysis between the differences and the mean of the measurements. The coefficient B was 0.08, and the non-significant *p*-value indicated that there is no systematic variation depending on the level of the measurement and thus differences between the two methods are stable.

#### 3.3.5. Bodygram Dashboard^®^ and FFM (DXA)

**Figure 5:**
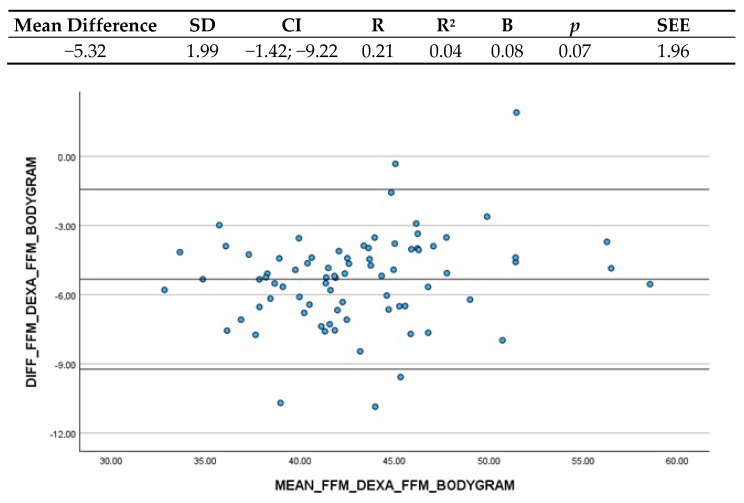
Bland–Altman plot and regression analysis between Bodygram Dashboard^®^ FFM estimation and ALST estimated by DXA. SD = standard deviation; CI = Confidence Interval; R = correlation coefficient; R^2^ = correlation coefficient squared; B = regression coefficient; *p* = significance; SEE = standard estimation error.

The comparison between the Bodygram Dashboard^®^ FFM estimation and FFM obtained through DXA yielded a mean difference of −5.32 kg (CI: −1.42; −9.22), indicating that BIA overestimated DXA measurements by more than 5 kg (Figure 5). The Confidence Interval resulted quite high, but still clinically acceptable. Concerning the linear regression, the coefficient B was 0.08, and the non-significant *p*-value indicated no systematic variations depending on the level of the measurement and thus differences between the two methods are stable.

## 4. Discussion

With the ultimate objective of making sarcopenia screening easier, faster, and more effective, this paper presented the results of a cross-sectional study aiming at comparing ASMM and FFM estimated through DXA and estimated through BIA. As the latter is much more manageable in settings where a wide range of populations can be assessed, comparisons among the already existing equations and DXA estimations made clearer which equation can better estimate ASMM and FMM in a general population of 40–70 year-old women.

Despite the first attempt to divide the sample by means of age and then by the presence of menopause, the comparison among groups did not yield enough significant differences, and the main result concerned the phase angle evaluated by BIA. Indeed, in both comparisons, this marker was higher in the younger population and in the sample with no menopause, indicating that muscle quality might be better in such groups. This result can be explained by the physiological trend of losing muscle mass with ageing and after menopause as well as the tendency for low chronic inflammation during the postmenopausal phase [11], in which extra-cellular water (ECW) increases, reducing PA. The reduction in PA with age is in line with the study of Campa et al. (2023) [21], in which PA centiles showed how this variable decreases during ageing, reducing muscle quality and increasing ECW. In concern to muscle quantity, only the DXA ALST estimation and Kyle et al.’s (2003) estimation [17] highlighted significant differences between women with menopause and with no menopause, indicating there could be an effective difference, as already shown by Rathnayake et al. (2023) [22] in a wider sample estimated by DXA. Despite that, mainly due to the different sample sizes of the two age groups, the researchers performed the analyses in the whole sample, finding that BIA yielded mostly comparable estimations with regard to DXA-estimated ASMM and FFM, confirming the usability of such a method in a non-clinical setting in middle-aged women.

Concerning the ASMM estimation equations, all showed great agreement with DXA estimations, with a global mean difference below 3 kg. The most suitable seemed to be Scafoglieri et al. (2017) [19], which yielded a mean difference of only 1.81 kg. This result fit very little with the context where such an equation was created, as it involved 291 people aged 70+ with functional limitations. Despite that, the DXA model was the same as the one we used in this study, and the majority of the sample was composed of women (70%). Scafoglieri and colleagues [19] concluded that “the proposed ALM equations seem to be more reliable when applied to sarcopenic older adults than the ones previously published in the literature [17,18], which were developed in adults without physical function decline”; meanwhile, we found out that, in our sample, probably due to the use of the same DXA model, such an equation can be also applied to younger women. Indeed, the main predictors of the equation were sex, weight, and Impedance Index, with no reference to age or functional ability. It is important to highlight that Kyle et al.’s (2003) [17] and Sergi et al.’s (2015) [18] equations, despite being created with different DXA models (Hologic), also showed highly comparable results, and their application for the screening of muscle mass can be considered clinically acceptable. Indeed, as Kyle et al.’s (2003) [17] equation was created using a wider and more heterogeneous sample (aged 20–99), we might expect a higher agreement, but probably, the difference in the DXA models impacted the analysis. Lastly, Sergi et al. (2015) [18] involved older people (aged 60+) and used the Hologic DXA model, yielding the highest difference in the performed comparisons (although still clinically acceptable). In light of the above-mentioned considerations, in the case of 40–70 year-old women, we suggest applying such equations, with a preference for Scafoglieri et al. (2017) [19], as all showed great accordance with DXA estimations.

In regard to FFM, Kanellakis et al. (2020) [20] yielded a very high accordance with DXA estimations, as the mean difference was just 0.5 kg. Indeed, this equation was created involving a population of more than 600 subjects. With regard to Bodygram Dashboard^®^, the equation used to estimate FFM is proprietary to Akern but equivalent to Sun et al.’s (2003) [23]. The mean difference was of −5.32 kg compared to DXA, which is greater than that of Kanellakis et al. (2020) [20]. This discrepancy can be attributed to the differing methodologies employed in the development of the two equations. The Akern equation was developed through a water-based molecular model, whereas the Kanellakis equation was developed based on a DXA-based anatomical tissue model.

With regard to limitations, the main issue is represented by the small sample size. Secondly, the presented research was limited only to a Northern Italian context.

## 5. Conclusions

This study showed how the use of BIA represents an effective and reliable method in the evaluation of sarcopenia. Moreover, this research highlighted how DXA estimations and the selected BIA estimation equations either for ASMM or for FFM were found to be in great agreement, in particular the one published by Scafoglieri et al. (2017) [19] for ASMM and Kanellakis et al.’s equation (2020) [20] for FFM. Thus, BIA, which is a faster, portable, and low-cost method, could be used in a screening setting (clinical and non-clinical) and possibly intercept low muscle mass conditions which can be linked to sarcopenia. Particular attention and focus in future studies should be pointed to the phase angle, which can yield precious information on muscle quality. Application ranges from clinical settings such as outpatient nutrition assessments to wider contexts such as public engagement events, where it is also possible to provide on-target lifestyle advice and address the subjects to specific treatment, involving either nutritional or physical activity. Indeed, the early detection of such status can lead to a prompt lifestyle intervention, involving a higher protein consumption and ad hoc physical activity in order to re-build and strengthen the lost muscle mass [24].

## Figures and Tables

**Table 1:** Descriptive statistics of the total sample and divided by the two targeted groups. Between groups comparisons were obtained through Independent Student’s *t*-test. Rz: Resistance; Xc: Reactance; PA: Phase Angle; ASMM: Appendicular Skeletal Muscle Mass estimated by BIA; ALST = Appendicular Lean Soft Tissue estimated by DXA; FFM = Fat-Free Mass estimated by BIA.

	Total Sample (n = 79)		40–55 Years Old (n = 38)		56–70 Years Old (n = 41)		*p*
	Mean	St. Dev.	Mean	St. Dev.	Mean	St. Dev	
**Age (years)**	55.68	7.94	48.84	4.68	62.02	4.13	**<0.001**
**Stature (cm)**	158.62	6.25	159.73	6.77	157.6	5.61	0.13
**Weight (kg)**	66.61	12.49	65.05	9.84	68.05	14.49	0.28
**BMI (kg/m^2^)**	26.59	5.37	25.62	4.4	27.49	6.05	0.12
**Rz (ohm)**	542.06	68.55	539.8	66.33	544.16	71.3	0.78
**Xc (ohm)**	51.7	6.58	52.87	6.32	50.62	6.7	0.12
**PA (°)**	5.48	0.58	5.63	0.63	5.34	0.49	**<0.05**
**FFM (DXA) (kg)**	40.41	5.26	40.87	5.27	39.99	5.28	0.46
**ALST (kg)**	19.29	2.87	19.61	2.85	19.01	2.88	0.35
**ASMM Lunar (Scafoglieri et al., 2017) [19] (kg)**	17.48	2.45	17.43	2.11	17.53	2.76	0.86
**ASMM (Kyle et al., 2003) [17] (kg)**	17.04	2.52	17.25	2.3	16.84	2.72	0.47
**ASMM (Sergi et al., 2015) [18] (kg)**	16.38	2.24	16.49	2.03	16.28	2.44	0.68
**FFM (Kanellakis et al., 2020) [20] (kg)**	39.89	4.86	40.4	4.52	39.43	5.16	0.38
**FFM Bodygram Dashboard (kg)**	45.73	4.86	46.04	4.51	45.45	5.2	0.60

**Table 2:** Descriptive statistics of sample and divided by the presence of menopause. Between-group comparisons were obtained through Independent Student’s *t*-test. Rz: Resistance; Xc: Reactance; PA: Phase Angle; ASMM: Appendicular Skeletal Muscle Mass estimated by BIA; ALST = Appendicular Lean Soft Tissue estimated by DXA; FFM = Fat-Free Mass estimated by BIA.

	Menopause (n = 56)		No Menopause (n = 23)		*p*
	Mean	St. Dev.	Mean	St. Dev.	
**Age (years)**	59.2	6	47.13	5.07	**<0.001**
**Stature (cm)**	157.94	5.91	160.29	6.86	0.13
**Weight (kg)**	66.65	12.43	66.5	12.91	0.96
**BMI (kg/m^2^)**	26.85	5.48	25.95	5.16	0.5
**Rz (ohm)**	550.05	68.1	522.61	67.14	0.11
**Xc (ohm)**	51.37	6.74	52.52	6.23	0.48
**PA (°)**	5.36	0.55	5.77	0.54	**<0.01**
**FFM (DXA) (kg)**	39.56	4.57	42.48	6.28	0.05
**ALST (kg)**	18.75	2.38	20.63	3.51	**<0.05**
**ASMM Lunar (Scafoglieri et al., 2017) [19] (kg)**	17.29	2.31	17.96	2.76	0.27
**ASMM (Kyle et al., 2003) [17] (kg)**	16.67	2.3	17.94	2.85	**<0.05**
**ASMM (Sergi et al., 2015) [18] (kg)**	16.1	2.06	17.06	2.55	0.08
**FFM (Kanellakis et al., 2020) [20] (kg)**	39.19	4.68	41.62	4.94	**<0.05**
**FFM Bodygram Dashboard (kg)**	45.09	4.44	47.31	5.53	0.06

**Table 3:** Correlations between DXA and BIA estimations in the total sample. ASMM = Appendicular Skeletal Muscle Mass estimated by BIA; ALST = Appendicular Lean Soft Tissue estimated by DXA; FFM = Fat-Free Mass estimated by BIA.

BIA	DXA	R	*p*
**ASMM (kg) Kyle et al. (2003)** [17]	ALST (kg)	0.88	**<0.001**
**ASMM (kg) Sergi et al. (2015)** [18]	ALST (kg)	0.88	**<0.001**
**ASMM (kg) Scafoglieri et al. (2017)** [19]	ALST (kg)	0.82	**<0.001**
**FFM (kg) Kanellakis et al. (2020)** [20]	FFM (kg)	0.92	**<0.001**
**FFM (kg) Bodygram Dashboard^®^**	FFM (kg)	0.93	**<0.001**

## Data Availability

The original contributions presented in the study are included in the article, further inquiries can be directed to the corresponding author.
